# [^68^Ga]Ga-PSMA-11 PET imaging as a predictor for absorbed doses in organs at risk and small lesions in [^177^Lu]Lu-PSMA-617 treatment

**DOI:** 10.1007/s00259-021-05538-2

**Published:** 2021-10-08

**Authors:** Steffie M. B. Peters, Regina Hofferber, Bastiaan M. Privé, Maarten de Bakker, Martin Gotthardt, Marcel Janssen, Frank de Lange, Constantijn H. J. Muselaers, Niven Mehra, J. Alfred Witjes, Pedro F. Costa, James Nagarajah, Mark W. Konijnenberg, Walter Jentzen

**Affiliations:** 1 Department of Medical Imaging, Radboud University Medical Center, P.O. Box 9101, 6500 HB Nijmegen, The Netherlands; 2 Department of Nuclear Medicine, Essen University Hospital, Essen, Germany; 3 Department of Urology, Radboud University Medical Center, Nijmegen, The Netherlands; 4 Department of Medical Oncology, Radboud University Medical Center, Nijmegen, The Netherlands; 5 Department of Radiology and Nuclear Medicine, Erasmus MC, Rotterdam, The Netherlands

**Keywords:** [^177^Lu]Lu-PSMA-617, Dosimetry, Radionuclide therapy, Prostate cancer, mHSPC, [^68^Ga]Ga-PSMA-11

## Abstract

**Introduction:**

Patient eligibility for [^177^Lu]Lu-PSMA therapy remains a challenge, with only 40–60% response rate when patient selection is done based on the lesion uptake (SUV) on [^68^Ga]Ga-PSMA-PET/CT. Prediction of absorbed dose based on this pre-treatment scan could improve patient selection and help to individualize treatment by maximizing the absorbed dose to target lesions while adhering to the threshold doses for the organs at risk (kidneys, salivary glands, and liver).

**Methods:**

Ten patients with low-volume hormone-sensitive prostate cancer received a pre-therapeutic [^68^Ga]Ga-PSMA-11 PET/CT, followed by 3 GBq [^177^Lu]Lu-PSMA-617 therapy. Intra-therapeutically, SPECT/CT was acquired at 1, 24, 48, 72, and 168 h. Absorbed dose in organs and lesions (*n* = 22) was determined according to the MIRD scheme. Absorbed dose prediction based on [^68^Ga]Ga-PSMA-PET/CT was performed using tracer uptake at 1 h post-injection and the mean tissue effective half-life on SPECT. Predicted PET/actual SPECT absorbed dose ratios were determined for each target volume.

**Results:**

PET/SPECT absorbed dose ratio was 1.01 ± 0.21, 1.10 ± 0.15, 1.20 ± 0.34, and 1.11 ± 0.29 for kidneys (using a 2.2 scaling factor), liver, submandibular, and parotid glands, respectively. While a large inter-patient variation in lesion kinetics was observed, PET/SPECT absorbed dose ratio was 1.3 ± 0.7 (range: 0.4–2.7, correlation coefficient *r* = 0.69, *p* < 0.01).

**Conclusion:**

A single time point [^68^Ga]Ga-PSMA-PET scan can be used to predict the absorbed dose of [^177^Lu]Lu-PSMA therapy to organs, and (to a limited extent) to lesions. This strategy facilitates in treatment management and could increase the personalization of [^177^Lu]Lu-PSMA therapy.

**Supplementary Information:**

The online version contains supplementary material available at 10.1007/s00259-021-05538-2.

## Introduction

Prostate cancer accounts for 20% of new cancers diagnosed every year. With a mortality rate of 10%, it is one of the most common causes of death worldwide [1–3]. Treatment options include local radiotherapy, surgery, or systemic treatments such as hormonal therapy or chemotherapy. For metastasized disease, prostate-specific membrane antigen (PSMA), a protein that is overexpressed in most prostate cancer cells [4–6], can also be used as a target for radionuclide therapy. In end-stage castrate-resistant metastatic prostate cancer (mCRPC) patients, [^177^Lu]Lu-PSMA-617 [7–19] and/or [^225^Ac]Ac-PSMA-617 [20–24] showed remarkable responses with, in general, a mild toxicity profile. Therefore, [^177^Lu]Lu-PSMA is now also translated to earlier stages such as to hormone-sensitive prostate cancer (HSPC) with encouraging results [25].

At present, high tumor uptake of [^68^Ga]Ga-PSMA-11, [^18^F]DCFPyL, or [^18^F]PSMA-1007 on positron emission tomography (PET) imaging is mandatory for PSMA radioligand therapy [26–30]. In some studies, PET standardized uptake value (SUV) on [^68^Ga]Ga-PSMA-PET has been shown to correlate with absorbed (radiation) dose in lesions and salivary glands in both mCRPC [31] and mHSPC patients [32], while other studies did not find this correlation [9, 18, 33]. Patients selected based on PET lesion SUV show a response rate of only 40–60% [7–19]. A potential improvement of patient selection has been suggested by the group of Hofman and colleagues by using both FDG-positive tumor volume and mean intensity of PSMA-avid tumor uptake [34]. An actual dose estimation based on the pre-therapeutic [^68^Ga]Ga-PSMA-PET could provide more accurate information on expected treatment response, since the calculations of the absorbed doses take into account tracer kinetics and are intrinsically corrected for factors such as partial volume effect occurring in particular for small tumors. Therefore, we hypothesize that patient selection could be improved if the pre-therapeutic [^68^Ga]Ga-PSMA-PET data were used to predict absorbed doses for the subsequent [^177^Lu]Lu-PSMA treatment.

In addition, pre-therapeutic evaluation of risk for organ toxicity is important in order to design a patient-specific treatment plan. It can prevent clinicians from exceeding threshold doses for radiation-related toxicity and it can potentially be used to apply higher therapeutic activities. To this end, mean SUV of organs on PET are not a suitable parameter to predict organ absorbed dose, mainly due to heterogeneity in the PET signal. However, modeling the organ absorbed doses based on the pre-therapeutic [^68^Ga]Ga-PSMA-PET imaging could provide a tool to assess organ toxicity after treatment.

Similar studies have been carried out using PET/CT imaging for an absorbed dose prediction after radionuclide therapy, mainly using ^124^I for prediction of organ-absorbed dose after ^131^I-therapy in thyroid cancer patients [35–39]. This methodology is based on the assumption that tracer kinetics for ^124^I and ^131^I are comparable, and cumulated activity derived from multi time point ^124^I-PET/CT can be translated to ^131^I-cumulated activity, thereby predicting organ absorbed dose after therapy. It is suggested that this approach can be used to design patient-specific treatment by respecting the organ threshold dose for radiation toxicity effects [39, 40].

To date, the use of [^68^Ga]Ga-PSMA-PET for an absorbed dose estimation of [^177^Lu]Lu-PSMA treatment has not been reported in the literature. This study aims to fill this gap by investigating the predictive value of a single time point pre-therapeutic [^68^Ga]Ga-PSMA-PET for absorbed dose after [^177^Lu]Lu-PSMA therapy in organs (kidneys, salivary glands, and liver) and tumor lesions. It relies on tissue-specific radioligand kinetics that will be derived from therapeutic imaging data with [^177^Lu]Lu-PSMA-SPECT, in combination with tracer uptake of a single time point pre-therapeutic [^68^Ga]Ga-PSMA-PET. The predicted absorbed doses were compared to actually delivered absorbed doses in therapy.

## Materials and methods

### Patient population

The study comprised 10 patients with low-volume hormone-sensitive prostate cancer who received [^177^Lu]Lu-PSMA for treatment of oligometastatic prostate cancer. It was approved by the Medical Review Ethics Committee Region Arnhem-Nijmegen and was registered on clinicaltrials.gov (NCT03828838). The trial was done in accordance to the principles of Good Clinical Practice and the Declaration of Helsinki. All subjects provided written informed consent before study entry. A comprehensive description of the patient population has been published before [25]. In short, HSPC patients with prostate-specific antigen (PSA) doubling time ≤ 6 months and ≤ 10 visible metastases on baseline [^68^Ga]Ga-PSMA-PET/CT, with at least one lesion ≥ 10 mm in diameter, were included. Normal renal and bone marrow functions were required (MDRD-GFR ≥ 60 ml/min, white blood cell count > 3.5 × 109.131/l, platelet count > 150 × 109.132/l and hemoglobin > 6 mmol/l). A detailed study flowchart can be found in Online Resource 1.

### Imaging and therapy

Patients received [^68^Ga]Ga-PSMA-11-PET/CT approximately 1 week prior to radioligand therapy. Imaging was performed 60 ± 10 min post-injection on a Biograph mCT system (Siemens Healthineers, Erlangen, Germany) scanning cranium to trochanter major. Patients received a therapeutic activity of 3 GBq (3057 ± 38 MBq) [^177^Lu]Lu-PSMA-617. This relatively low activity was chosen because it was part of a prospective pilot study in a patient population that did not receive this type of treatment before. The preparation of [^177^Lu]Lu-PSMA was described previously [25] and can be found in Online Resource 2. SPECT/CT imaging was performed at 1, 24, 48, 72, and 168 h after administration on either a Symbia T16 or Symbia Intevo Bold system (Siemens Healthineers, Erlangen, Germany). SPECT/CT scans were acquired at three body regions: pelvis, abdomen, and head-neck regions. Acquisition and reconstruction parameters can be found in Online Resource 3.

### Organ and tumor volumes

Organ and tumor volumes were derived by manual segmentation (VOI technique) using the reference CT image. Some structures could not be reliably delineated on CT. As an alternative, volumes of the paired parotid and submandibular glands as well as bone lesions were determined using a PET-based iterative thresholding method [41]. It has been shown that this technique allows for accurate volume estimation down to the ^68^Ga-PET spatial resolution of 0.13 ml and reveals reliable volume estimates for objects with moderate non-uniform activity distributions.

### Imaged SPECT and PET activities in organs and lesions and their corrections

To determine the SPECT and PET activities in organs, a contour-based approach was applied: the imaged activity, *A*_ContourVOI_, within the contour of the organ boundary was determined and corrected for partial volume effects using the fitted isovolume recovery coefficient, *RC*_iso_. The corrected organ activity *A*_Corrected_ is given as follows:

1 $${A}_{\mathrm{Corrected}}=\frac{{A}_{\mathrm{ContourVOI}}}{{RC}_{\mathrm{iso}}}$$

The isovolume *RC* values depend on spatial resolution and patient’s individual organ volumes [42].

The diameters of the lesions were small (median diameter of 12 mm, range: 6–43 mm), and thus clearly below the SPECT spatial resolution of 15 mm. For a tissue size smaller than 1.25–1.5 times the spatial resolution, correction for partial volume effect is not recommended [42, 43]. Instead, an oversize-based method was applied [44–46]. An oversized lesion VOI was drawn large enough to include the entire tail of the lesion activity, and its cross-contamination from the surrounding background activity was further removed using the following equation:

2 $${A}_{\mathrm{Corrected}}={A}_{\mathrm{OversizeVOI}}-\left({V}_{\mathrm{OversizeVOI}}-{V}_{\mathrm{Lesion}}\right)\cdot {C}_{\mathrm{Background}}$$

where *A*_Corrected_ is the corrected lesion activity, *V*_Lesion_ is the lesion volume, *A*_OversizeVOI_ is the imaged activity of the oversized lesion VOI, and *c*_Background_ is the background activity concentration derived from a representative background VOI close to the lesion. Of note, the drawback of this oversized-based approach is that high and non-uniform background may result in activity underestimation.

### Biokinetic analysis—parametrization of the tissue-specific radioligand uptake curves

**Fig. 1:**
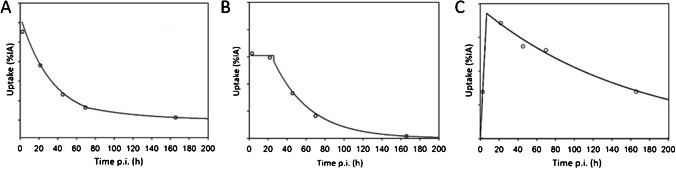
Types of uptake pattern depending on tissue type. **A** Segmented bi-exponential uptake curve for liver and kidneys. **B** Rectangular mono-exponential uptake curve for salivary glands. **C** Linear mono-exponental curve for lesions

The observed tissue-specific [^177^Lu]Lu-PSMA uptake curves were analyzed to derive a typical time-activity curve (TAC) or, equivalently, uptake curves for each organ and for the lesions. First, to avoid ambiguity and maintain consistency for all uptake curves, no curve-fitting procedure was applied. Hence, the common approach to fit the 5 data points at once using, for instance, a bi-exponential function, was not used. Instead of fitting the entire dataset, the biokinetic data were segmented into three phases, that is, an early, a mid, and a late phase to extract the typical uptake pattern of each tissue type within a time segment. Second, the exploratory investigation of the intra-therapeutic uptake curves revealed that there are three types of uptake patterns. Figure 1 schematically illustrates the 3 phases and the respective piecewise parameterization of representative uptake curves. For kidneys and liver, an instantaneous uptake (early phase) followed by a mono-exponential clearance with an effective half-life *T*_eff,1_ up to 72 h (mid-phase), and thereafter, a second mono-exponential clearance (late phase) with an effective half-life *T*_eff,2_ (Fig. 1A) was observed. Thus, the early and mid-phases were parameterized using the half-life *T*_eff,1_ and the late phase was parameterized using half-life *T*_eff,2_; the respective half-lives were obtained by linear regression analyses. For the salivary glands, kinetics was assessed for the whole organ instead of separate glands. An instant uptake was observed, and its value remained almost constant with a (average) value of *U*_0_ up to 24 h (early and mid-phases). More precisely, the 24 h value was sometimes above or below the 1 h uptake value; the average uptake value was used to effectively represent these phases. Thereafter, a mono-exponential clearance with an effective half-life *T*_eff_ (late phase) was found (Fig. 1B). For lesions, a linear increase (early phase) was observed with a slope derived from the first uptake value *U*(*t*_1_), that is, *α* = $\frac{U(1)}{{t}_{1}}$ (in %/h), to a maximum uptake *U*_max_ (mid-phase) followed by a mono-exponential decay (late phase) with an effective half-life *T*_eff_ (Fig. 1C), with the intercept of both functions at time *t*_max_. For clarity, the respective equations are given in Online Resource 4.

The mean values of the intra-therapeutic kinetic parameters were calculated and used to predict the biokinetics for each tissue type based on a single PET-based uptake value of [^68^Ga]Ga-PSMA.

### Pre-therapeutic [^177^Lu]Lu-PSMA dosimetry using [^68^Ga]Ga-PSMA SPECT/CT imaging

For the absorbed dose prediction, a correction regarding the differences in the physical half-lives of ^68^Ga (*T*_Ga_) and ^177^Lu (*T*_Lu_) is necessary according to the radioactive decay law. The observed [^68^Ga]Ga-PSMA uptake value *U*_Ga_(*t*_PET_) was projected to the predicted [^177^Lu]Lu-PSMA uptake value *U*_Lu_(*t*_PET_) using the following equation [47]:

3 $${U}_{\mathrm{Lu}}\left({t}_{\mathrm{PET}}\right)={U}_{\mathrm{Ga}}({t}_{\mathrm{PET}})\cdot \mathrm{exp}(\frac{\mathrm{ln}\left(2\right)}{{T}_{\mathrm{Ga}}}\cdot {t}_{\mathrm{PET}})\cdot \mathrm{exp}(-\frac{\mathrm{ln}(2)}{{T}_{\mathrm{Lu}}}\cdot {t}_{\mathrm{PET}})$$

This projected uptake value was used to construct the individual uptake curve based on the tissue-specific biokinetic model, from which the projected [^177^Lu]Lu-PSMA residence times (or TIAC values) were estimated. In the construction of the projected uptake curve, the tissue-specific mean values of the intra-therapeutic kinetic parameters were applied. The projection of the functions to determine the TIAC values is given in Online Resource 5.

For each organ and lesion, the projected [^177^Lu]Lu-PSMA TIAC and the mass were used to predict the absorbed dose per unit administered [^177^Lu]Lu-PSMA activity using Olinda 2.2.

### Software and statistical analysis

The image interpolation and image analyses were conducted using PMOD 4.2 software (PMOD Technologies Ltd., Zurich, Switzerland). Statistical analysis was performed using GraphPad Prism 5.03 (Graphpad Software Inc., CA, USA). The descriptive statistics included the mean, median, standard deviation (SD), and range and were expressed in the following form: mean ± SD (median, minimum–maximum). Uncertainty in the absorbed dose values were determined following the EANM uncertainty guideline by Gear et al. (for more details see Online Resource 6). Differences between the 2 groups were evaluated by the Mann–Whitney *U* test. A Spearman non-parametric correlation test was used to evaluate correlations between lesion SUV_max_ on [^68^Ga]Ga-PSMA-PET and absorbed dose on [^177^Lu]Lu-PSMA-SPECT, between lesion-absorbed dose based on [^68^Ga]Ga-PSMA-PET and [^177^Lu]Lu-PSMA-SPECT, and between lesion PET/SPECT absorbed dose ratio and lesion volume. A *p*-value of less than 0.05 was considered to be statistically significant.

## Results

### General

Patient characteristics and administered activities (GBq) can be found in Online Resource 7. A total of 22 lesions were evaluated (1 to 7 per patient). For 8 lesions, volume determined on CT was 6.5 ± 14.6 ml (0.9, 0.21–42.5, Table 1). For the other 14 lesions, volume was determined on PET with a volume of 3.0 ± 5.3 ml (1.1, 0.13–20.2). In order to compare the methodology, volume was determined on both CT and PET for 9 lesions. This showed a mean ratio of 1.09 ± 0.17 for PET volume versus CT volume. The difference in volume was not significant (Mann–Whitney *U* test: *p* = 0.86).

**Table 1:** Biokinetics of [^177^Lu]Lu-PSMA for each lesion. Slope *α* of initial uptake phase; *t*_max_ time to maximum uptake, *U*_max_; effective half-life *T*_eff_ of mono-exponential decay phase between *t*_24h_ and *t*_168h _

Patient number	Tumor number^a^	Volume (ml)	Tissue type	*α* (%/h)	*t*_max_ (h)	*T*_eff_ (h)
2	1	0.50	Bone	0.001	9	137
	2	0.61	Bone	0.036	5	114
	3	3.48	Bone	-^b^	-^b^	58
3	1	1.45	Lymph node	0.026	3	67
4	1	0.13	Lymph node	0.031	2	94
	2	0.43	Bone	0.059	3	63
	4	2.80	Lymph node	0.005	3	85
	5	2.98	Bone	0.337	4	70
	6	7.52	Bone	0.020	3	87
	7	20.21	Bone	0.128	3	60
	9	42.49	Lymph node	0.006	2	63
5	1	0.57	Lymph node	-^b^	-^b^	67
	3	0.66	Lymph node	0.012	4	78
	5	0.72	Lymph node	-^b^	-^b^	51
6	1	0.68	Lymph node	0.012	7	57
	2	1.05	Lymph node	0.005	4	98
7	1	0.19	Lymph node	-^b^	-^b^	123
	2	2.68	Lymph node	-^b^	-^b^	96
8	1	1.61	Lymph node	0.075	3	60
9	1	0.21	Lymph node	0.023	4	75
	2	0.70	Lymph node	-^b^	-^b^	128
10	1	2.00	Bone	0.014	5	65
Median (range)		0.89 (0.13–42.49)		0.021 (0.001–0.337)	3 (2–9)	72 (51–137)
Mean ± SD		4.26 ± 9.56		0.049 ± 0.081	4 ± 2	82 ± 25

^a^Lesion numbering is a result of initial region drawing; therefore, missing numbers do not represent excluded lesions. ^b^For these lesions, uptake on the 1-h time point [^177^Lu]Lu-PSMA-SPECT was not visible; therefore, α and *t*_max_ could not be assessed

**Fig. 2:**
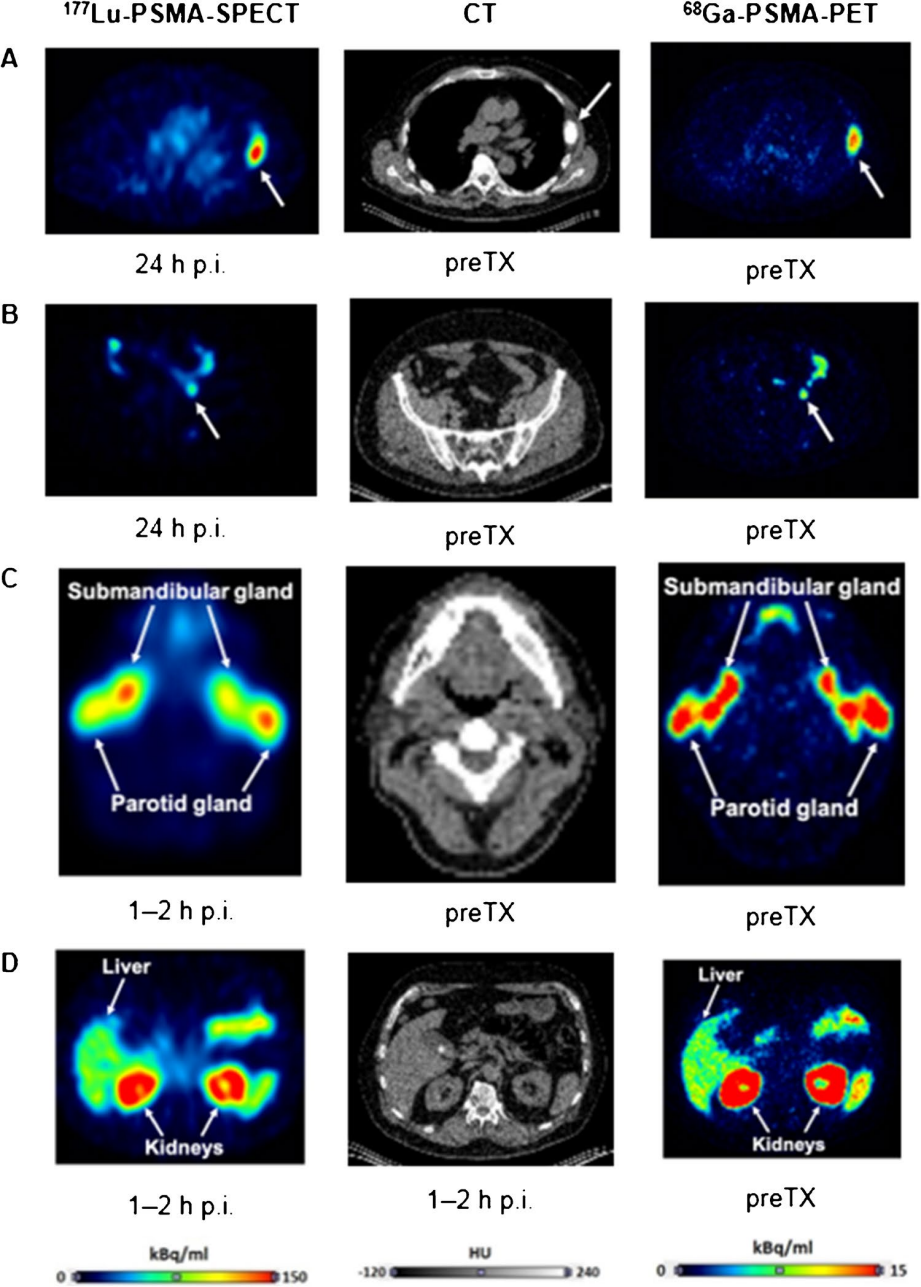
Representative images of [^177^Lu]Lu-PSMA-SPECT (left), CT (center), and [^68^Ga]Ga-PSMA-PET (right) for lesions and organs. **A** Bone lesion in abdomen region (visible on CT). **B** Lymph node lesion in pelvis region (not visible on CT). **C** salivary glands. **D** Kidneys and liver. The time points are chosen to visualize maximum uptake

Figure 2 shows an example of typical images for the organs of interest and two lesions for [^177^Lu]Lu-PSMA-SPECT, CT, and [^68^Ga]Ga-PSMA-PET. For the lesions, it shows that some are clearly visible on CT (Fig. 2A) while others are not (Fig. 2B), while both lesions in this example are clearly visible and delineable on PET as well as SPECT.

### Analysis of the intra-therapeutic biokinetics

Uptake curves were determined based on the effective half-lives for each organ (Table 2) and lesions (Table 1). Kidneys showed a median half-life of 28 h and 49 h for the first and second excretion phases, respectively. Liver showed a median half-life of 21 h and 47 h for the first and second excretion phases, respectively. For the salivary glands, uptake between *t*_0_ and *t*_24h_ was assumed to be constant based on the average uptake of U_1h_ and U_24h_ (Fig. 1B). For the second phase after 24 h, median half-life was 33 h.

**Table 2:** Biokinetics of [^177^Lu]Lu-PSMA for the organs of each patient

Patient number	Effective half-life *T*_eff_ (h)				
	Kidneys		Liver		Salivary glands
	0–72 h	72 h-∞	0–72 h	72 h-∞	24 h-∞
1	35	47	18	58	31
2	40	50	23	42	32
3	29	47	21	48	34
4	24	49	21	47	35
5	34	49	23	42	31
6	26	44	20	47	30
7	25	46	18	45	32
8	31	49	21	54	37
9	26	53	23	42	34
10	21	50	19	48	34
Median (range)	28 (21–40)	49 (44–53)	21 (18–23)	47 (42–58)	33 (30–37)
Mean ± SD	29 ± 6	49 ± 2	21 ± 2	47 ± 5	33 ± 2

 For 6 out of the 22 lesions, the volume was not visible on the 1-h time point [^177^Lu]Lu-PSMA-SPECT; therefore, the initial uptake kinetics could not be determined individually. Instead, uptake phase was estimated by taking the mean *t*_max_ for the other 16 lesions, which was 3.9 h. Median effective half-life T_eff_ for clearance after 24 h was 72 h. Of note, kinetics were not significantly different between bone and lymph node lesions (Mann–Whitney *U*-test: *p* = 0.86).

### Intra-therapeutic and predicted absorbed doses for organs

**Fig. 3:**
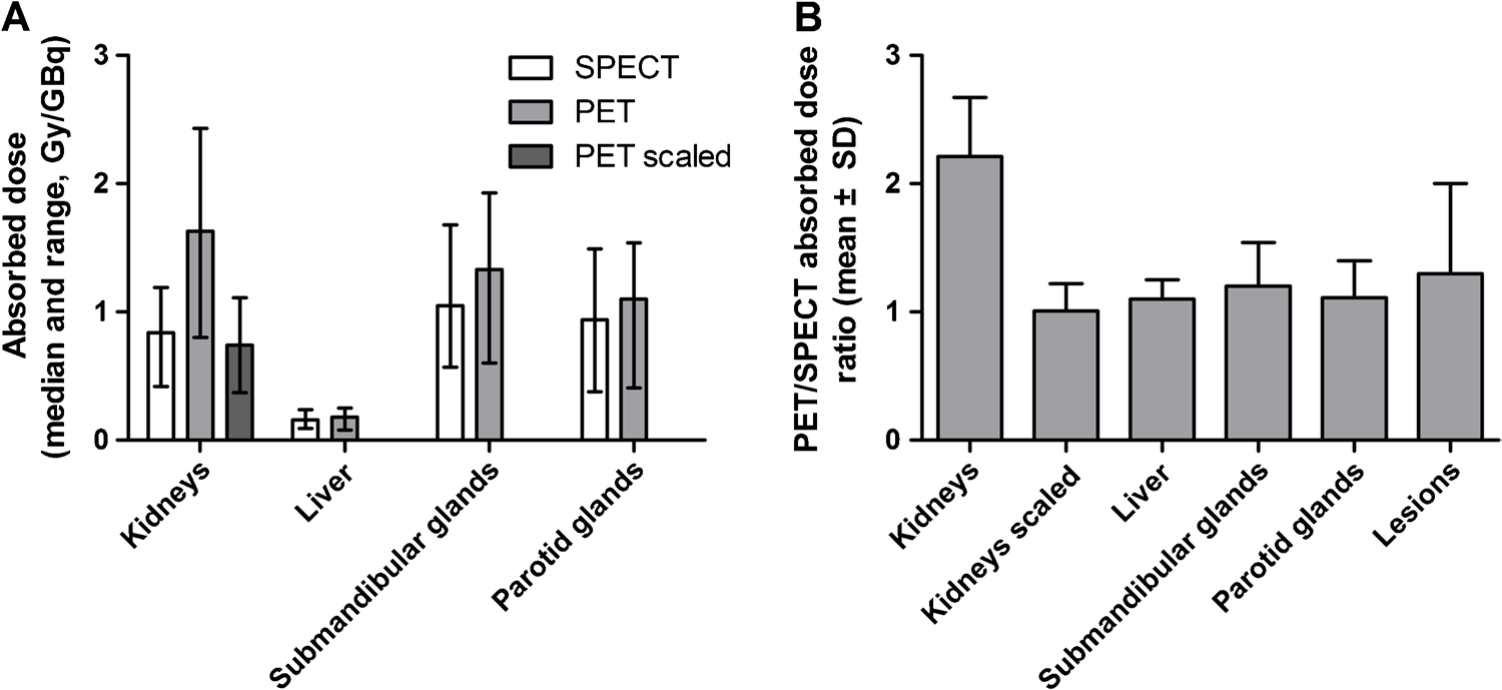
Absorbed dose per organ as determined from [^68^Ga]Ga-PSMA-PET and [^177^Lu]Lu-PSMA-SPECT. **A** Median and range. **B** PET/SPECT absorbed dose ratio for the organs. The kidney absorbed dose ratio corrected with a scaling factor *F* = 2.2 is shown as well

The median absorbed dose as determined from [^177^Lu]Lu-PSMA-SPECT as well as the median predicted absorbed dose from [^68^Ga]Ga-PSMA-PET can be found per organ in Fig. 3. Combined statistics and organ kinetics per patient can be found in Online Resources 8–11. For the kidneys, initial PET/SPECT absorbed dose ratio was 2.21 ± 0.46 (1.32–2.75). Because the ratio was rather constant, a scaling factor *F* = 2.2 was introduced by which the PET-predicted absorbed dose was divided, leading to a PET/SPECT absorbed dose ratio for the kidney was 1.01 ± 0.21 (0.60–1.25). For liver, submandibular glands, and parotid glands, agreement between SPECT and PET absorbed dose was high, with PET/SPECT absorbed dose ratios of 1.10 ± 0.15 (0.94–1.35), 1.20 ± 0.34 (0.61–1.84), and 1.11 ± 0.29 (0.54–1.47), respectively. No scaling factor was introduced for these organs.

### Intra-therapeutic and predicted absorbed doses for lesions

**Fig. 4:**
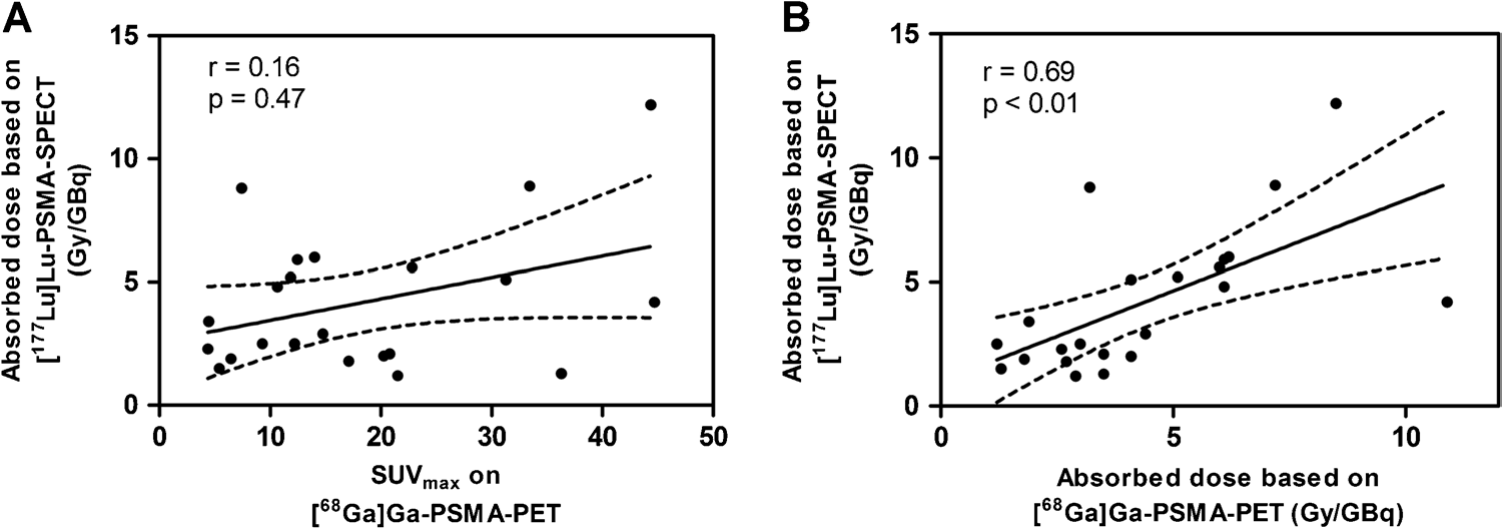
Correlation between lesion SUV_max_ on [^68^Ga]Ga-PSMA-PET and absorbed dose based on [^177^Lu]Lu-PSMA-SPECT (**A**), and between absorbed dose as determined from [^68^Ga]Ga-PSMA-PET and [^177^Lu]Lu-PSMA-SPECT (**B**). Dotted lines indicate 95% confidence intervals

**Fig. 5:**
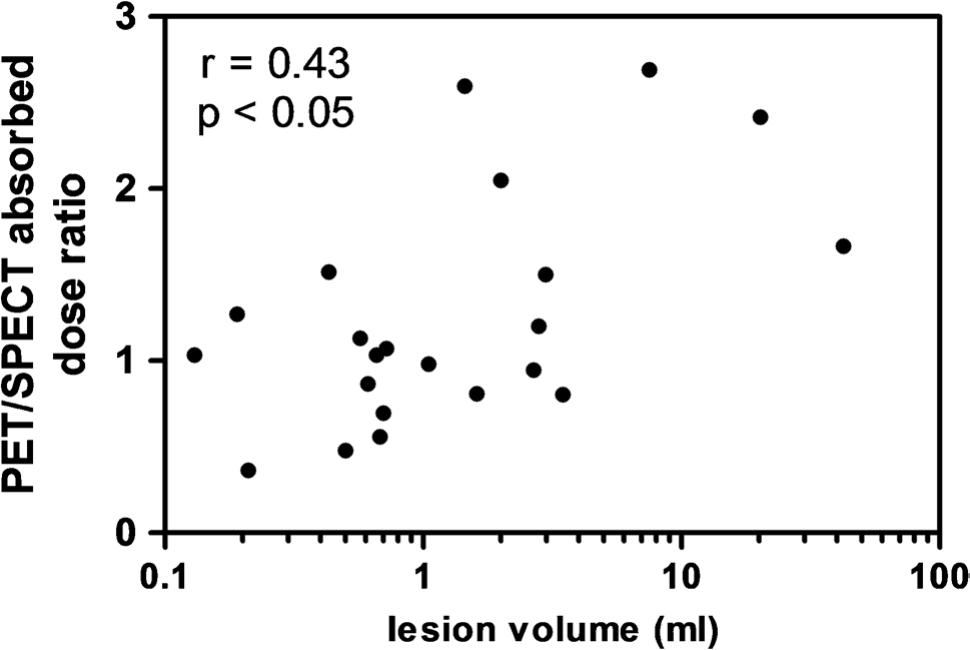
PET/SPECT absorbed dose ratio as a function of lesion volume

The results per lesion can be found in Table 3 (SUV_max_ on [^68^Ga]Ga-PSMA-PET, as well as absorbed dose predicted from [^68^Ga]Ga-PSMA-PET and determined from [^177^Lu]Lu-PSMA-SPECT). The PET/SPECT absorbed dose ratio for lesions was 1.3 ± 0.7 (1.1, 0.4–2.7). No significant correlation was found between SUV_max_ on [^68^Ga]Ga-PSMA-PET and absorbed dose from [^177^Lu]Lu-PSMA-SPECT (*r* = 0.16, *p* = 0.47, Fig. 4A), while a significant correlation was found between predicted absorbed dose from [^68^Ga]Ga-PSMA-PET and determined absorbed dose from [^177^Lu]Lu-PSMA-SPECT (*r* = 0.69, *p* < 0.01, Fig. 4B). Lesion volume dependency of PET/SPECT absorbed dose ratio is shown in Fig. 5, where it can be seen that an underestimation of absorbed dose was mainly found for the smaller lesion volumes (Spearman significant correlation, *r* = 0.43, *p* < 0.05).

**Table 3:** Overview of results per lesion: SUV_max_ on [^68^Ga]Ga-PSMA-PET, predicted absorbed dose from [^68^Ga]Ga-PSMA-PET, and absorbed dose determined from [^177^Lu]Lu-PSMA-SPECT

Patient number	Tumor number		SUV_max_ (PET)	PET AD^1^ (Gy/GBq) ± error	SPECT AD^a^ (Gy/GBq) ± error
2	1		9.3	1.2 ± 0.4	2.5 ± 0.3
	2		31.3	4.1 ± 1.3	5.1 ± 0.5
	3		5.4	1.3 ± 0.3	1.5 ± 0.2
3	1		44.7	10.9 ± 3.6	4.2 ± 0.7
4	1		12.2	3.0 ± 1.0	2.5 ± 0.6
	2		36.3	3.5 ± 1.2	1.3 ± 0.1
	4		14.8	4.4 ± 1.5	2.9 ± 0.5
	5		20.8	3.5 ± 1.1	2.1 ± 0.2
	6		17.1	2.7 ± 0.9	1.8 ± 0.3
	7		21.5	2.9 ± 1.0	1.2 ± 0.2
	9		14.0	6.2 ± 2.1	6.0 ± 0.7
5	1		12.5	6.1 ± 2.0	5.9 ± 0.9
	3		22.8	6.0 ± 2.0	5.6 ± 0.7
	5		4.4	2.6 ± 0.8	2.3 ± 0.3
6	1		11.8	5.1 ± 1.7	5.2 ± 0.8
	2		4.5	1.9 ± 0.6	3.4 ± 0.4
7	1		11.7	6.1 ± 2.0	4.8 ± 1.2
	2		6.5	1.8 ± 0.6	1.9 ± 0.2
8	1		33.4	7.2 ± 2.4	8.9 ± 0.9
9	1		44.4	8.5 ± 2.8	12.2 ± 1.6
	2		7.4	3.2 ± 1.1	8.8 ± 1.3
10	1		20.3	4.1 ± 1.4	2.0 ± 0.2
Median (range)			14.4 (4.4–44.7)	3.8 (1.2–10.9)	3.2 (1.2–12.2)
Mean ± SD			18.5 ± 12.4	4.4 ± 2.4	4.2 ± 2.9

^a^*AD*, absorbed dose

## Discussion

This study evaluated the possibility to use a single time point [^68^Ga]Ga-PSMA-PET scan to predict the therapeutic absorbed dose in organs at risk and lesions for a subsequent treatment with [^177^Lu]Lu-PSMA therapy. Tracer kinetics is a crucial part in these predictive absorbed dose calculations, which determines the shape of the uptake time-activity curve and thus the cumulated activity. In this study, these kinetics were determined as the mean kinetics of 10 patients based on the SPECT data. This means that this approach is based on two main assumptions: firstly, the typical shape of the uptake curves for organs and lesions for the different patients are nearly identical; therefore, it is justified to use general tissue-specific kinetics in the PET prediction model. Secondly, the different tracers used in PET and SPECT imaging (PSMA-11 and PSMA-617, respectively) have similar kinetics; therefore, the kinetics found for PSMA-617 on SPECT can be used to project the expected kinetics of PSMA-11 on PET. Multiple studies investigated biodistribution and kinetics for PSMA-11 [48–51] and PSMA-617 [52] and showed indeed similar kinetic behavior [53].

Prediction of absorbed dose for lesions showed a large variation in kinetics between patients both during the uptake phase (SD of 50%) and the excretion phase (SD of 30%), indicating that the first assumption of identical lesion kinetics between patients does not hold. Therefore, tumor lesion dosimetry using a single time [^68^Ga]Ga-PSMA-PET was challenging. Earlier studies found that different tracer kinetics could be the result of different lesion types (bone versus lymph node lesions) [54]. However, in our study no statistically significant difference in tracer kinetics between the two tissue types was found (*p* = 0.84). The highly variable kinetics observed in lesions are possibly the result of heterogeneity in tumor biology. Therefore, the use of a general tracer uptake pattern for lesions will introduce relevant deviations on an individual level.

However, the proposed methodology using lesion-specific kinetics results in a rather good PET/SPECT absorbed dose ratio for lesions of 1.3 ± 0.7 (0.4–2.7), with a significant correlation (*r* = 0.69, *p* < 0.01) that was not found between SUV_max_ on [^68^Ga]Ga-PSMA-PET and absorbed dose after therapy (*r* = 0.16, *p* = 0.47). So, despite a relatively large range in PET/SPECT absorbed dose ratio, an actual absorbed dose prediction could still mean a significant improvement in patient selection compared to only using lesion SUV_max_, since it provides better insight in what lesion uptake is to be expected and thus whether treatment with [^177^Lu]Lu-PSMA is expected to be effective.

Estimation of patient-specific tracer uptake in lesions could potentially be improved by obtaining continuous information on tracer distribution during the first hour after injection of [^68^Ga]Ga-PSMA using dynamic PET imaging [54]. Moreover, obtaining uptake information at multiple later time points could provide crucial information on late tracer kinetics, which largely determine the absorbed dose. However, due to the short half-life of ^68^Ga (68 min), it is not possible to follow the retention of PSMA over multiple days. The positron emitter ^89^Zr with a 3.27 days half-life could be an attractive alternative. The first preclinical studies with [^89^Zr]Zr-PSMA-617 and [^89^Zr]Zr-PSMA-I&T biodistribution showed that this resembled the distribution of [^177^Lu]Lu-PSMA-617 and [^177^Lu]Lu-PSMA-I&T, respectively (data not published yet). Recently the first clinical study showed that several lesions had uptake on [^89^Zr]Zr-PSMA-PET, which were not detected on early time point PET using ^18^F-FDG or [^68^Ga]Ga-PSMA [55]. Therefore, ^89^Zr-labelled PSMA has the potential to improve lesion absorbed dose prediction.

The large range in PET/SPECT absorbed dose ratio found in this study can also partly be explained by difficulties in calculating SPECT absorbed dose for small structures, such as the lesions found in this patient cohort. Due to limited image resolution, count statistics, and photon scatter, determination of residence times is difficult. This means that in general, larger uncertainties in absorbed dose calculations are found in these small volumes [32, 56].

While patient selection might be improved by combining lesion SUV on PSMA-PET with evaluation of positive tumor uptake on ^18^F-FDG-PET [34], this does not provide information on risk of organ toxicity. The mHSPC patient cohort for this study, acute organ toxicity, is not anticipated, since these patients tend to have a relatively good physical condition and good organ function. However, development of chronic toxicities should be prevented. In addition, presently, [^177^Lu]Lu-PSMA therapy is mainly applied in mCRPC patients, which are at risk for compromised organ function and may have received prior radionuclide therapy that already deposited a radiation dose to the healthy organs. Therefore, an absorbed dose prediction based on the pre-therapeutic [^68^Ga]Ga-PSMA-PET scan would provide the physician with a useful tool to manage or refrain from additional treatment cycles when there is a significant risk of organ toxicity. Our study showed that an absorbed dose prediction based on a single time point [^68^Ga]Ga-PSMA-PET scan is feasible, similar to what earlier studies found for ^124^I-PET/CT dose prediction of ^131^I-therapy in thyroid cancer patients [35–40]. Tissue-specific organ kinetics showed to be stable between patients, which means that uptake information at a single time point in combination with assumed tissue-specific tracer kinetics provide an effective instrument for absorbed dose prediction. Although it was shown earlier that organ tracer kinetics in mHSPC patients are very similar to those in mCRPC patients [32], it would be advised to establish tissue-specific tracer kinetics for mCRPC patients when applying the proposed methodology in this specific patient group. Furthermore, our results are based on only 10 patients. More elaborate data of larger patient cohorts is warranted.

Initially, we found that the absorbed dose prediction based on PET for the kidneys was notably higher than the SPECT-based values: PET/SPECT absorbed dose ratio of 2.21 ± 0.46 (Fig. 3B). A possible explanation could be a difference in early phase kinetics between patients, which was the only exception found in this study that showed somewhat larger variation in tracer kinetics: 21% for the early phase kinetics up to 72 h (Table 2). In addition, there might be a difference in 1-h tracer uptake between PSMA-11 and PSMA-617. Since the PSMA tracer is cleared mainly via the kidneys, a potential faster blood and renal uptake for PSMA-11 would lead to a higher activity found in the kidney at 1 h p.i. on PET than for PSMA-617 at the same time point on SPECT. In addition, there are some differences in the coordination chemistry of [^68^Ga]Ga-PSMA-11 and [^177^Lu]Lu-PSMA-617; Ga^3+^ forms a hexadentate binding in the HBED chelator leaving two nitrogens and Lu^3+^ a octadentate binding in the DOTA chelator. In preclinical setting, it was shown that this leads to a higher kidney uptake of [^68^Ga]Ga-PSMA-11 in comparison to [^111^In]In-PSMA-617 [57, 58]. This would then lead to an overestimation of the total predicted absorbed dose. A remarkable feature was that the PET/SPECT absorbed dose ratio for kidney was rather constant. After applying a scaling factor of 2.2, a PET/SPECT absorbed dose ratio of 1.01 ± 0.21 (Fig. 3B) was obtained. Thus, despite an initial overestimation of kidney absorbed dose based on [^68^Ga]Ga-PSMA-PET, it can reliably be used to predict therapeutic absorbed dose for [^177^Lu]Lu-PSMA after applying the scaling factor, with a maximum deviation of around 20%. For the other organs, such a deviation was not found so no scaling was performed. However, the salivary glands showed a relatively large range in PET/SPECT absorbed dose ratio for the submandibular glands (0.61–1.84) and parotid glands (0.54–1.47), respectively (Fig. 3). This indicates that, despite very comparable overall tracer kinetics in the salivary glands, the uptake at 1 h p.i. can be rather variable between patients, leading to a larger range in PET/SPECT ratio.

## Conclusion

This study showed that a single time point [^68^Ga]Ga-PSMA-PET scan can be used to predict the absorbed dose of [^177^Lu]Lu-PSMA therapy to the kidney, liver, salivary glands, and (to a limited extent) to tumor lesions. The proposed methodology is readily available for clinical implementation since the pre-treatment PSMA-PET scan is already required for [^177^Lu]Lu-PSMA therapy. This strategy facilitates in treatment management and could increase the personalization of [^177^Lu]Lu-PSMA therapy.

## Supplementary Information

Below is the link to the electronic supplementary material.

Supplementary file1 (DOCX 136 KB)

## Data Availability

The datasets generated during and/or analyzed during the current study are available from the corresponding author on reasonable request.

## Abbreviations

CT: Computed tomography
DLP: Dose length product
FWHM: Full width at half maximum
mCRPC: Metastasized castrate-resistant prostate cancer
MIRD: Medical internal radiation dose
HSPC: Hormone-sensitive prostate cancer
OP-OSEM-TOF: Ordinary Poisson ordered-subset expectation maximization with time-of-flight
OSEM: Ordered-subset expectation maximization
PET: Positron emission tomography
p.i.: Post-injection
PSA: Prostate-specific antigen
PSMA: Prostate-specific membrane antigen
RC: Recovery coefficient
SD: Standard deviation
SPECT: Single-photon emission computed tomography
SUV: Standardized uptake value
TAC: Time-activity curve
TIAC: Time-integrated activity coefficient
VOI: Volume of interest
