# Supplementary material for: [68Ga]Ga-PSMA-11 PET imaging as a predictor for absorbed doses in organs at risk and small lesions in [177Lu]Lu-PSMA-617 treatment
Source: Eur J Nucl Med Mol Imaging. 2021 Oct 8;49(4):1101–12. doi: 10.1007/s00259-021-05538-2 (PMC8921092; doi:10.1007/s00259-021-05538-2)
Supplement: Supplementary file 1 — Supplementary file1 (DOCX 136 KB) [file 259_2021_5538_MOESM1_ESM.docx]

Supplementary materials


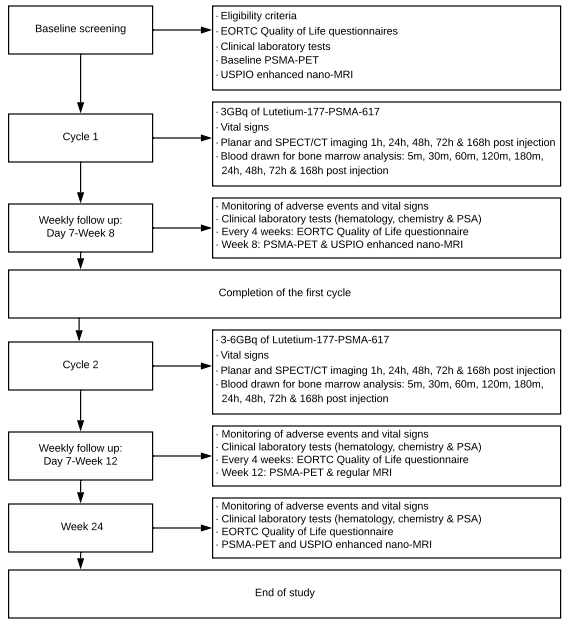


Online Resource 1: Study flowchart

**Online Resource 2: Labeling and purification of PSMA-617 with ^177^Lu**

^177^LuCl_3_ was obtained from ITG (Garching, Germany). GMP-grade PSMA-617 was obtained from ABX (Radeberg, Germany). The radiolabeling of PSMA-617 was performed on GRP synthesis module (Scintomics, Fürstenfeldbruck, Germany) using sterile and GMP-grade SC-105 kits. In brief, 4 mg gentisic acid and the PSMA-617 peptide were dissolved in 500 μL WFI and added to the reaction vessel. After addition of the ^177^LuCl_3_ in sodium acetate buffer and ascorbic acid the reaction was incubated at 100 °C for 20 minutes. After cooling down, the product was diluted to 16.5 ml with saline/DTPA to which 0.9 ml ethanol has been added. The radioactive solution was filtered through a 0.22 μm filter (Millex GV. Merck, Amsterdam, The Netherlands) and dispensed into a closed glass type I container. Microbiological monitoring in class C was performed during synthesis, filtration and dispensing. Assembling of the dispensing and filtration system was performed in a class A isolator with a class B airlock (in a class C background). The radiolabeled PSMA-617 was measured for total radioactivity in a calibrated activity dose calibrator prior to injection and injected within 6 hours after radiolabeling.

**Online Resource 3: Imaging protocols**

**[^68^Ga]Ga-PSMA -11-PET/CT**

Patients received [^68^Ga]Ga-PSMA -11-PET/CT prior to radioligand therapy. Imaging was performed 60 ± 10 minutes post-injection on a Biograph mCT system (Siemens Healthineers, Erlangen, Germany) scanning cranium to trochanter major. For the pelvis region, data were acquired using 4 minutes per bed position, whereas 3 minutes per bed position were applied for the other regions. PET data were reconstructed using ordinary Poisson ordered-subset expectation maximization with time-of-flight modelling (OP-OSEM-TOF) with 2 iterations and 21 subsets, matrix size of 400 (resulting in a cuboid-shape voxel size of approximately 2.0 mm^3^) and a smoothing Gaussian filter of 3 mm. The estimated reconstructed PET spatial resolution expressed as full width at half maximum (FWHM) was 6.3 mm. A low dose CT was performed (average dose length product (DLP) of 291 mGy·cm) and the CT data were reconstructed using 3.0 mm slice thickness and kernel B19f; the reconstructed transverse CT images had a voxel size of 1.0 x 1.0 x 3.0 mm^3^. For all PET images, standard corrections for CT-based attenuation, scatter, decay, and dead-time were performed.

**[^177^Lu]Lu-PSMA SPECT/CT**

Patients received a therapeutic activity of 3 GBq (3057 ± 38 MBq) [^177^Lu]Lu-PSMA. SPECT/CT imaging was performed at 1, 24, 48, 72 and 168 hours after administration on either a Symbia T16 or Symbia Intevo Bold system (Siemens Healthineers, Erlangen, Germany). SPECT/CT scans were acquired at three body regions: the pelvis, abdomen, and head-neck region (64 projections per detector, time per projection of 14 s, a 20% photon energy window at 208 keV, and dual-energy window for Compton scattering. SPECT data were reconstructed using OSEM reconstruction (Flash 3D with collimator detector response) using 4 iterations and 8 subsets, matrix size of 128 (resulting in a cuboid-shape voxel size of 4.8 mm^3^) and a smoothing Gaussian filter of 8.4 mm. The estimated reconstructed SPECT spatial resolution was 15 mm (FWHM). A low dose CT was performed (average DLP of 130 mGy·cm ) and the data were reconstructed using B31s kernel and 3.0 mm slice thickness resulting in a voxel size of 1.0 x 1.0 x 3.0 mm^3^. Of note, the SPECT image reconstruction approach takes into account corrections for scatter, CT-based attenuation, and dead-time

**Online Resource 4: Parametrization of the uptake curves depending on tissue type**

For kidneys and liver, a mono-exponential clearance with an effective half-life *T*_eff,1_ up to 72 h and thereafter a second mono-exponential clearance with an effective half-life *T*_eff,2_ was observed (Figure 1A). Mathematically, the uptake curve is given as:

For salivary glands, kinetics was assessed for the whole organ instead of separate glands. An instant uptake was observed and its value remained almost constant with an (average) value of *U*_0_ up to 24 h. Thereafter, a mono-exponential clearance with an effective half-life *T*_eff_ was found (Figure 1B), according to

For lesions, a linear increase was observed with a slope, α = $\frac{U(1)}{t_{1}}$ (in %/h), to a maximum uptake, *U*_max_, followed by a mono-exponential decay with an effective half-life *T*_eff_ (Figure 1C), with the intercept of both functions at time *t*_max_,according to:

**Online Resource 5: Projections of the functions to estimate the TIAC** **based solely PET uptake**

The projection of the functions given in Online Resource 10 led to the following expressions for TIAC in each organ or lesion based solely on the projected PET uptake *U*_Lu_(*t*_PET_). In the following, the uptake value *U*_Lu_(*t*_PET_) given in the equations are non-percentage values:

**Online Resource 6: Uncertainty analysis**

**Data collection:**

- 1. Quantitative SPECT at 5 time-points, within 45 minute scan-time
  2. Quantitative PET at 1 time point
  3. Organ specific tracer kinetics based on PET

**Uncertainty in SPECT cumulated activity**

1. Drawing of VOIs in SPECT data over organs and tumor lesions to determine counts:
2. Large spherical VOI over lesions with background correction; error: 10%
3. CT based VOI over organs; error 5%
4. Fit to Time-Activity Curve on SPECT
5. Mono-exponential fit between activity on each subsequent SPECT time point, considered an approximation of trapezoid integration
6. Integration over time of the TAC, to determine the time-integrated activity Ã on SPECT (reference absorbed dose)
7. Trapezoid integration method; error:

$$\left[ \frac{\delta\tilde{A}}{\tilde{A}} \right]^{2}=\sum_{i} \left[ \frac{\delta A(t_{i})}{A(t_{i})} \right]^{2}$$

**Uncertainty in PET cumulated activity**

1. Drawing of VOIs in PET data over organs and tumor lesions to determine counts. Error in counts δSUV
2. Large spherical VOI over lesions with background correction; error: 10%
3. CT based VOI over organs; error 5%
4. Determination of average organ specific tracer kinetics based on SPECT. Uncertainty δk is based on the SD of the patient average kinetics
   1. Lesions: 30%
   2. Organs: 10%
5. Determination of PET time-integrated activity Ã. Error:

$$\left[ \frac{\delta\tilde{A}}{\tilde{A}} \right]^{2}={\left[ \frac{\delta SUV}{SUV} \right]^{2}+\left[ \frac{\delta k}{k} \right]}^{2}$$

**General uncertainty**

1. Error in SPECT camera specific calibration factor for ^177^Lu: 5% [Peters, 2020]

CF = 10.6 ± 0.5 cps/MBq

1. Determination of lesion and organ volume:
2. Lesion and organ volume based on PET/CT and diagnostic CT [Jentzen, 2015];

Voxelisation and resolution error volume with lesion diameter d and voxel size a: $\left[ \frac{\delta V}{V} \right]^{2}=9 \left[ \frac{\delta d}{d} \right]^{2}=1.5\left( \frac{a}{d} \right)^{2}$ [Gear, 2018]

1. Determine volume specific lesion S-factor with power-function on S-values spheres$S=81.7V^{-0.988}$; error:

$$\left[ \frac{\delta S}{S} \right]^{2}=\left( 0.988\frac{\delta V}{V} \right)^{2}$$

1. Absorbed dose calculation with MIRD equation $D=\tilde{A}\times S$; error:

$$\left[ \frac{\delta D}{D} \right]^{2}={\left[ \frac{\delta\tilde{A}}{\tilde{A}} \right]^{2}+\left[ \frac{\delta S}{S} \right]}^{2}$$

Online Resource 7: patient characteristics and administered activities

| Patient # | Age  (yrs) | Weight  (kg) | Administerd activity (GBq) |
| --- | --- | --- | --- |
| 1 | 61 | 89 | 3.0 |
| 2 | 62 | 91 | 3.1 |
| 3 | 77 | 77 | 3.1 |
| 4 | 66 | 96 | 3.1 |
| 5 | 68 | 78 | 3.0 |
| 6 | 65 | 86 | 3.0 |
| 7 | 71 | 75 | 3.1 |
| 8 | 71 | 59 | 3.1 |
| 9 | 69 | 90 | 3.0 |
| 10 | 62 | 85 | 3.0 |

Online Resource 8: Individual kidney volumes and absorbed dose from ^177^Lu-PSMA-SPECT and ^68^Ga-PSMA-PET

| Patient nr. | Kidney volume (ml) | | Absorbed dose (Gy/GBq)^a^ ± error | | |
| --- | --- | --- | --- | --- | --- |
|  | Right | Left | SPECT | PET unscaled | PET scaled^b^ |
| 1 | 183 | 200 | 0.42 ± 0.04 | 0.80 ± 0.09 | 0.37 ± 0.04 |
| 2 | 213 | 196 | 0.88 ± 0.03 | 1.16 ± 0.14 | 0.53 ± 0.06 |
| 3 | 184 | 201 | 0.67 ± 0.01 | 1.70 ± 0.20 | 0.77 ± 0.09 |
| 4 | 186 | 179 | 0.57 ± 0.05 | 1.56 ± 0.18 | 0.71 ± 0.08 |
| 5 | 157 | 151 | 0.80 ± 0.07 | 2.18^c^ ± 0.26 | 0.99 ± 0.12 |
| 6 | 187 | 187 | 0.51 ± 0.04 | 1.41 ± 0.17 | 0.64 ± 0.08 |
| 7 | 183 | 164 | 1.10 ± 0.10 | 2.43 ± 0.29 | 1.11 ± 0.13 |
| 8 | 149 | 148 | 1.03 ± 0.03 | 2.41 ± 0.29 | 1.10 ± 0.13 |
| 9 | 200 | 253 | 0.88 ± 0.13 | 1.49 ± 0.18 | 0.68 ± 0.08 |
| 10 | 164 | 175 | 1.19 ± 0.09 | 2.35 ± 0.28 | 1.07 ± 0.13 |
| Mean ± SD | 181 ± 19 | 185 ± 29 | 0.81 ± 0.25 | 1.75 ± 0.54 | 0.80 ± 0.25 |
| Median (range) | 184  (149 – 213) | 183  (148 – 253) | 0.84  (0.42 – 1.19) | 1.63  (0.80 – 2.43) | 0.74  (0.37 – 1.11) |

^a^: Absorbed dose was determined as the mean absorbed dose for the left and right kidney. ^b^: determined as the product between the absorbed dose for PET unscaled and scaling factor S = 2.21. ^c^: Pre-treatment ^68^Ga-PSMA-PET not available, therefore absorbed dose was determined on post-treatment ^68^Ga-PSMA-PET.

Online Resource 9: Individual liver volumes and absorbed dose from ^177^Lu-PSMA-SPECT and ^68^Ga-PSMA-PET

| Patient nr. | Liver volume (ml) | Absorbed dose (Gy/GBq) ± error | |
| --- | --- | --- | --- |
|  |  | SPECT | PET |
| 1 | 2382 | 0.091 ± 0.003 | 0.086 ± 0.01 |
| 2 | 1902 | 0.17 ± 0.01 | 0.16 ± 0.02 |
| 3 | 1979 | 0.15 ± 0.01 | 0.20 ± 0.02 |
| 4 | 1809 | 0.15 ± 0.01 | 0.18 ± 0.02 |
| 5 | 1799 | 0.17 ± 0.01 | 0.18^a^ ± 0.02 |
| 6 | 1798 | 0.15 ± 0.01 | 0.15 ± 0.02 |
| 7 | 1627 | 0.15 ± 0.01 | 0.20 ± 0.02 |
| 8 | 1185 | 0.24 ± 0.01 | 0.25 ± 0.02 |
| 9 | 1862 | 0.18 ± 0.01 | 0.17 ± 0.02 |
| 10 | 1513 | 0.18 ± 0.02 | 0.21 ± 0.02 |
| Mean ± SD | 1786 ± 296 | 0.16 ± 0.04 | 0.18 ± 0.04 |
| Median (range) | 1804  (1185 – 2382) | 0.16  (0.091 – 0.24) | 0.18  (0.086 – 0.25) |

^a^: Pre-treatment ^68^Ga-PSMA-PET not available, therefore absorbed dose was determined on post-treatment ^68^Ga-PSMA-PET.

Online Resource 10: Individual parotid gland volumes and absorbed dose from ^177^Lu-PSMA-SPECT and ^68^Ga-PSMA-PET

| Patient nr. | Parotid gland volume (ml) | | Absorbed dose (Gy/GBq)^a^ ± error | |
| --- | --- | --- | --- | --- |
|  | Right | Left | SPECT | PET |
| 1 | 61 | 47 | 0.81 ± 0.04 | 1.09 ± 0.13 |
| 2 | 27 | 26 | 0.70 ± 0.02 | 0.89 ± 0.11 |
| 3 | 26 | 23 | 1.15 ± 0.04 | 1.22 ± 0.15 |
| 4 | 31 | 37 | 0.75 ± 0.07 | 1.11 ± 0.13 |
| 5 | 16 | 19 | 1.49 ± 0.06 | 1.54^b^ ± 0.19 |
| 6 | 40 | 38 | 1.27 ± 0.04 | 1.22 ± 0.15 |
| 7 | 26 | 30 | 0.74 ± 0.03 | 0.41 ± 0.05 |
| 8 | 20 | 23 | 1.24 ± 0.05 | 1.60 ± 0.19 |
| 9 | 27 | 28 | 1.06 ± 0.05 | 0.80 ± 0.10 |
| 10 | 40 | 39 | 0.38 ± 0.01 | 0.54 ± 0.06 |
| Mean ± SD | 31 ± 12 | 31 ± 9 | 0.96 ± 0.32 | 1.04 ± 0.37 |
| Median (range) | 27  (16 – 61) | 29  (19 – 47) | 0.94  (0.38 – 1.49) | 1.10  (0.41 – 1.60) |

^a^: Absorbed dose was determined as the mean absorbed dose for the left and right parotid gland. ^b^: Pre-treatment ^68^Ga-PSMA-PET not available, therefore absorbed dose was determined on post-treatment ^68^Ga-PSMA-PET.

Online Resource 11: Individual submandibular gland volumes and absorbed dose from ^177^Lu-PSMA-SPECT and ^68^Ga-PSMA-PET

| Patient nr. | Submandibular gland volume (ml) | | Absorbed dose (Gy/GBq)^a^ ± error | |
| --- | --- | --- | --- | --- |
|  | Right | Left | SPECT | PET |
| 1 | 17 | 14 | 0.57 ± 0.03 | 1.05 ± 0.13 |
| 2 | 11 | 11 | 1.30 ± 0.05 | 1.49 ± 0.18 |
| 3 | 9 | 7 | 1.00 ± 0.04 | 1.35 ± 0.17 |
| 4 | 17 | 16 | 0.93 ± 0.09 | 1.29 ± 0.16 |
| 5 | 10 | 11 | 1.57 ± 0.06 | 1.93^b^ ± 0.24 |
| 6 | 14 | 12 | 1.48 ± 0.06 | 1.31 ± 0.16 |
| 7 | 10 | 11 | 0.98 ± 0.04 | 0.60 ± 0.07 |
| 8 | 11 | 12 | 1.10 ± 0.05 | 1.50 ± 0.18 |
| 9 | 9 | 10 | 1.68 ± 0.08 | 1.35 ± 0.17 |
| 10 | 13 | 12 | 0.92 ± 0.03 | 1.25 ± 0.15 |
| Mean ± SD | 12 ± 3 | 12 ± 2 | 1.15 ± 0.33 | 1.31 ± 0.32 |
| Median (range) | 11  (9 – 17) | 11  (7 – 16) | 1.05  (0.57 – 1.68) | 1.33  (0.60 – 1.93) |

^a^: Absorbed dose was determined as the mean absorbed dose for the left and right submandibular gland. ^b^: Pre-treatment ^68^Ga-PSMA-PET not available, therefore absorbed dose was determined on post-treatment ^68^Ga-PSMA-PET.
